# The Distribution of Nitric Oxide‐Synthesizing Neurons and Soluble Guanylate Cyclase in Relation to Dopaminergic Systems in the Pigeon Brain

**DOI:** 10.1002/cne.70095

**Published:** 2025-10-15

**Authors:** Alina Steinemer, Marie Ziegler, Kevin Haselhuhn, Onur Güntürkün, Noemi Rook

**Affiliations:** ^1^ Department of Biopsychology, Institute of Cognitive Neuroscience, Faculty of Psychology Ruhr University Bochum Bochum Germany

**Keywords:** dopaminergic pathways, memory flexibility, NADPH, nidopallium caudolaterale, nitric oxide, tyrosine hydroxylase

## Abstract

Nitric oxide (NO) is a diffusible neuromodulator that acts as a retrograde messenger in vertebrates, regulating synaptic plasticity and interacting with dopaminergic systems. Despite its established functions in mammals, little is known about the anatomical distribution and potential roles of NO in birds. Using histochemical and immunohistochemical techniques, we mapped NO‐synthesizing neurons—identified by NADPH‐diaphorase (NADPH‐d) and neuronal nitric oxide synthase (nNOS)—and analyzed their relationship with soluble guanylate cyclase (sGC) and tyrosine hydroxylase (TH)‐positive dopaminergic pathways in the pigeon brain. We found NADPH‐d labeling throughout forebrain, midbrain, and hindbrain regions. Within dopaminergic midbrain structures, the locus coeruleus showed strong colocalization with nNOS, and moderate overlap was observed in the ventral tegmental area, substantia grisea centralis, and substantia nigra. In the nidopallium caudolaterale (NCL), a prefrontal‐like associative area, sGC‐positive neurons were contacted by both TH‐ and NADPH‐d–positive fibers. Our results highlight an anatomical link between NO and dopaminergic pathways in pigeons, suggesting that mechanisms of memory flexibility known from other species may also operate in birds. In particular, our findings support the potential for NO–dopamine interactions in avian species, reminiscent of recently described mechanisms in *Drosophila melanogaster*. In this invertebrate model, a subset of dopaminergic neurons was shown to co‐release NO, targeting sGC‐expressing neurons to modulate memory flexibility, a circuit‐level organization not yet described in vertebrates

## Graphical abstract

NO‐synthesizing neurons, soluble guanylate cyclase, and catecholaminergic inputs converge in the pigeon nidopallium caudolaterale, suggesting a substrate for nitric oxide–dopamine interactions in memory flexibility.

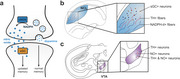

**Abbreviations**
Ac: nucleus accumbens
AD: dorsal arcopallium
AI: intermediate arcopallium
AL: ansa lenticularis
BC: brachium conjunctivum
BCD: brachium conjunctivum descendens
BCTS: brachium conjunctivum descendens et tractus tectospinalis
BSTL: lateral bed nucleus of the stria terminalis
CDL: area corticoidea dorsolateralis
CHCS: tractus cortico‐habenularis et cortico septalis
CoS: nucleus commissuralis septi
CPi: piriform cortex
CPP: prepiriform cortex
CS: nucleus centralis superior
D: nucleus of Darkschewitsch
DAB: 3,3‐diaminobenzidine
DLd: dorsal portion of dorsolateral area of the hippocampus
DLv: ventral portion of the dorsolateral area of the hippocampus
DMd: dorsal portion of dorsomedial area of the hippocampus
DMv: ventral portion of the dorsomedial area of the hippocampus
E: entopallium
EPL: external plexiform layer
EW: nucleus of Edinger‐Westphal
FRL: formation reticularis lateralis mesencephalic
FRM: formation reticularis medialis mesencephalic
GCt: substantia grisea centralis
GL: glomerular layer
GLdp: nucleus geniculatus lateralis, pars dorsalis principalis
GLv: nucleus geniculatus lateralis, pars ventralis
GP: globus pallidus
HA: hyperpallium apicale
HD: hyperpallium densocellulare
HI: hyperpallium intercalatum
HM: nucleus habenularis medialis
ICo: intercollicular complex
IGL: internal granular layer
Imc: nucleus isthmi, pars magnocellularis
IO: nucleus isthmo‐opticus
IP: nucleus interpeduncularis
Ipc: nucleus isthmi, pars parvocellularis
IS: nucleus interstitialis
LA: nucleus lateralis anterior thalami
LC: nucleus linearis caudalis
LFB: lateral forebrain bundle
LFS: lamina frontalis superior
LHy: area lateralis hypothalami
LLd: nucleus lemnisci lateralis, pars dorsalis
LoC: locus ceruleus
LSt: lateral striatum
M: mesopallium
MCL: mitral cell layer
MLd: nucleus mesencephalicus lateralis, pars dorsalis
MPv: nucleus mesencephalicus profundus, pars ventralis
MSt: medial striatum
N: nidopallium
NADPH: reduced form of nicotinamide adenine dinucleotide phosphate
NADPH‐d: NADPH diaphorase
nBOR: nucleus of the basal optic root
NCC: nidopallium caudocentrale
NCL: nidopallium caudolaterale
NDB: nucleus of the diagonal band
NIII: nervus occulomotorius
NIL: nidopallium intermedium laterale
NIM: nidopallium intermedium medialis
nIV: nucleus nervi trochlearis
nNOS: neuronal nitric oxide synthase
NO: nitric oxide
OB: olfactory bulb
OM: tractus occipitomesencephalicus
Pap: nucleus papilloformis
PBS: phosphate‐buffered saline
PBST: phosphate‐buffered saline with Triton X‐100
PFA: paraformaldehyde
PL: nucleus pontis lateralis
PM: nucleus pontis medials
PoA: posterior pallial amygdala
POA: nucleus preopticus anterior
QF: tractus quintofrontalis
RPgc: nucleus reticularis pontis caudalis, pars gigantocellularis
RPO: nucleus reticularis pontis oralis
Rt: nucleus rotundus
Ru: nucleus ruber
sGC: soluble guanylate cyclase
SL: nucleus septalis lateralis
SLu: nucleus semilunaris
SM: nucleus septalis medialis
SN: substantia nigra
SOp: stratum opticum
SpM: nucleus spiriformis medialis
TeO: tectum opticum
TH: tyrosine hydroxylase
TnA: nucleus taeniae of the amygdala
TPO: temporo‐parieto‐occipital area
Tr: triangular area of the hippocampus
TSM: tractus septo‐mesencephalicus
TuO: tuberculum olfactorium
Vl: lateral portion of the V‐shaped area of the hippocampus
VLV: nucleus ventmtis lemnisci lateralis
Vm: medial portion of the V‐shaped area of the hippocampus
VP: ventral pallidum
VTA: ventral tegmental area

## 1 Introduction

In ever‐changing environments, animals must adapt their memories to ensure survival, employing mechanisms such as extinction learning (Packheiser et al. 2021). Recent discoveries in *Drosophila melanogaster* have revealed an elegant mechanism supporting such memory flexibility: a subset of dopaminergic neurons releases both dopamine and nitric oxide (NO), enabling the fly to both reinforce and gradually “forget” memories associated with rewards or punishments (Aso et al. 2019; Green and Lin 2020). In this system, dopamine strengthens memory traces tied to meaningful experiences, while NO serves as a complementary, slowly acting signal that weakens these memories over time. This combination of reinforcement and decay ensures that memories remain adaptable—an essential trait for navigating dynamic surroundings. Interestingly, some neurons release dopamine but not NO, creating a heterogeneous ensemble of neurons that keep up the trace for different lengths of time.

The striking role of NO in memory adaptability in flies raises an intriguing question: could vertebrate brains also rely on NO for similar purposes? In vertebrates, nitric oxide is widely recognized as a retrograde messenger that regulates presynaptic transmitter release, contributes to synaptic plasticity and learning, and has established functional links with dopaminergic signaling (Pogun et al. 1994). In mammals, NO further influences learning, memory formation, neurogenesis, and neuroprotection (Bon and Garthwaite 2003; Zoubovsky et al. 2011). NO is produced from l‐arginine by neuronal nitric oxide synthase (nNOS) in a process requiring reduced nicotinamide adenine dinucleotide phosphate (NADPH) as a cofactor (Förstermann and Sessa 2012). As a diffusible gas, NO acts on various molecular targets, with its most significant effects mediated through soluble guanylate cyclase (sGC), which transduces NO signals to downstream pathways involved in neuronal function (Krumenacker et al. 2004).

Most knowledge of NO distribution in the brain comes from studies on human and rodent tissue, leaving NO distribution in birds less well understood. Nonetheless, several avian species have been investigated for NO‐synthesizing neurons, including Japanese quail (*Coturnix japonica* [Panzica et al. 1994]), rock pigeon (*Columba livia* [Atoji et al. 2001]), budgerigar (*Melopsittacus undulates* [Cozzi et al. 1996]), and chicken (*Gallus domesticus* [Brüning 1993]). Across these studies, NO‐synthesizing neurons were found in a variety of telencephalic, thalamic, midbrain, hindbrain, and cerebellar areas. However, species differences were noted, particularly in regions like the hippocampus and olfactory bulb (Atoji et al. 2001). In zebra finches (*Taeniopygia guttata*), investigations focused specifically on NO‐synthesizing neurons within the song system (Wallhäusser‐Franke et al. 1995), indicating specialized roles in vocal learning.

In contrast to the relatively well‐documented NO synthesis, detailed information on the distribution of the NO receptor molecule sGC is only available for rodents (Ariano et al. 1982; Ding et al. 2004; Furuyama et al. 1993) and has not yet been explored in any avian species. This gap in avian research also leaves us without insights into the potential colocalization of NO‐synthesizing neurons with sGC, although such colocalization has been observed in rats (Ding et al. 2004; Schmidt et al. 1992).

Furthermore, evidence from rodents indicates significant interactions between NO and dopaminergic systems. For example, a notable number of neurons in the rat ventral tegmental area co‐express both NO and dopamine (Klejbor et al. 2004), a pattern similarly found in the human temporal cortex (Benavides‐Piccione and DeFelipe 2003). These findings align with earlier studies suggesting NO's role in modulating dopamine release and uptake (Kiss et al. 1999; Pogun et al. 1994). Although comparable studies in birds have not yet been conducted, a similar NO–dopamine relationship may exist within avian species as well. This lack of information leaves an open question: could birds also exhibit a NO‐mediated mechanism for memory flexibility akin to that in fruit flies (Aso et al. 2019; Green and Lin 2020)?

To address this, the present study investigates the distribution of NO‐synthesizing neurons in the pigeon brain, focusing on their colocalization with tyrosine hydroxylase (TH), an enzyme critical in dopamine synthesis. Moreover, we examined the colocalization of NO‐, TH‐, and sGC‐synthesizing structures in prefrontal areas that might be related to memory flexibility. By exploring whether these NO pathways overlap with dopaminergic systems, we aim to determine if a similar neuroanatomical framework for adaptive memory processing is present in avian species.

Due to its gaseous nature, NO cannot be visualized directly; therefore, we employed immunohistochemical stainings of nNOS and histochemical stainings of NADPH‐diaphorase (NADPH‐d), widely used markers for NO‐synthesizing neurons (Suárez et al. 2006). This combined approach not only clarifies NO's anatomical distribution in the pigeon brain but also allows us to assess whether NO–dopamine interactions that support memory flexibility in fruit flies might have an evolutionary basis in vertebrates as well. Findings from this study could provide significant insights into conserved mechanisms of memory dynamics across species, offering a broader understanding of how neural systems support flexible memory.

## 2 Methods

### 2.1 Experimental Subjects

This study included a total of 16 homing pigeons (*Columba livia*), from which four were used for a combined staining of TH and NADPH‐d, two for a triple staining of NADPH‐d, TH, and sGC, six for a combined staining of nNOS and TH, and four for a single NADPH‐d staining. In accordance with the 3R principles of ethical animal research—Replacement, Reduction, and Refinement—this study was conducted using brain tissue from pigeons that had been perfused as part of unrelated, ethically approved experiments. No animals were sacrificed specifically for the purposes of this study. All animals were obtained from local breeders and were individually housed in wire‐mesh cages. Housing rooms were controlled for temperature, humidity, and day/night cycles (12‐h light–dark cycle). All experiments were performed according to the guidelines regarding the care and use of animals adopted by the German Animal Welfare Law for the prevention of cruelty to animals in agreement with the Directive 2010/63/EU of the European Parliament and of the Council of September 22 2010, and were approved by the animal ethics committee of the Landesamt für Natur, Umwelt und Verbraucherschutz NRW, Germany. All efforts were made to minimize the number of animals used and to minimize their suffering.

### 2.2 Perfusion and Tissue Processing

For perfusion, animals received an intramuscular injection of 0.1 mL heparin (B. Braun, Melsungen, Germany) diluted in 0.1 mL of 0.9% sodium chloride (NaCl) to prevent blood clots during perfusion. After a dwell time of 10 min, equithesin (0.45 mL/100 g body weight) was injected either intramuscularly into the breast muscle or intravenously into the brachial vein. As soon as both eyelid closure and toe pinching reflexes were negative and the heart stopped beating, animals were transcardially perfused with 500 mL of 0.9% NaCl (40°C), followed by ice‐cold (4°C) 4% paraformaldehyde (PFA) in 0.12 M phosphate‐buffered saline (PBS; pH 7.4). After that, the fixated brains were dissected and incubated in a postfix solution (30% sucrose in 4% PFA) for 2 h at 4°C. For cryoprotection, brains were transferred into sucrose solution (30% sucrose in PBS) for 24 h. Subsequently, brains were embedded in 15% gelatin dissolved in 30% sucrose solution for optimal stereotactic alignment and again fixated in 4% PFA solution for 24 h. Embedded brains were then transferred into 30% sucrose solution for at least 24 h. Finally, brains were cut into 40‐µm‐thick slices in frontal plane using a rotating microtome (Leica, Wetzlar, Germany). Slices were collected in 10 series per bird and stored in storage buffer (0.1% sodium azide in PBS) at 4°C until further processing.

### 2.3 Histochemistry and Immunohistochemistry

For all stainings, every tenth slice of a brain was used, and all procedures were conducted with free floating slices. First, we aimed to visualize TH, a precursor of dopamine, in combination with NADPH‐d activity, which is colocalized with nNOS in brain tissue (Dawson et al. 1991; Hope et al. 1991; Saffrey et al. 1992; Tracey et al. 1993; Young et al. 1997). We conducted a 3,3‐diaminobenzidine (DAB) reaction first to visualize TH, followed by an incubation in nitroblue tetrazolium and NADPH to visualize NADPH‐d activity (Wallhäusser‐Franke et al. 1995). Slices were first rinsed in PBS (3 × 10 min) and then incubated in 0.3% hydrogen peroxide (H_2_O_2_) in distilled water for 30 min to block endogenous peroxidases. After that, slices were rinsed again (3 × 10 min in PBS) and incubated in 10% normal horse serum (Vectastain Elite ABC kit, Vector Laboratories, Newark, USA; Cat#: PK‐6102) in PBS containing 0.3% Triton‐X‐100 (PBST) for 60 min to block unspecific binding sites. Slices were transferred to the primary antibody solution containing a mouse anti‐TH antibody (1:1000 in PBST; Invitrogen, Darmstadt, Germany; Cat#: MA1‐24654, RRID: AB_795666) overnight at 4°C. On the next day, slices were again rinsed (3 × 10 min in PBS) and then incubated in the secondary antibody solution containing a biotinylated horse anti‐mouse antibody (1:500 in PBST; Vectastain Elite ABC kit, Vector Laboratories, Newark, USA; Cat#: PK‐6102) for 60 min at room temperature. Subsequently, slices were rinsed (3 × 10 min in PBS) and transferred to an avidin–biotin–peroxidase solution (1:100 in PBST each; Vectastain Elite ABC kit, Vector Laboratories, Newark, USA; Cat#: PK‐6102) for 60 min. After further rinsing (3 × 10 min in PBS followed by 1 × 5 min in 0.1 M sodium acetate buffer, pH 6.0), slices were incubated in DAB solution, which was composed of DAB (0.2 mg/mL), cobalt chloride (0.4 mg/mL), ammonium chloride (0.4 mg/mL), and β‐d‐glucose (4 mg/mL) in 0.1 M sodium acetate buffer. Contrary to our standard DAB protocol, we did not add ammonium nickel sulfate here in order to obtain a brown instead of a black reaction product that could be differentiated more easily from the following nitroblue NADPH‐d staining. The DAB reaction was initiated by adding glucose oxidase (80−100 µL/50 mL DAB solution), which led to the oxidation of the β‐d‐glucose. During the reaction, staining intensity was visually controlled continuously, and the solution was changed every 10 min. The staining reaction was stopped after 30 min by rinsing the slices in 0.1 M sodium acetate buffer (3 × 5 min) and PBS (3 × 5 min). For the following NADPH‐d staining, slices were incubated in a solution containing 1 mM NADPH (ThermoFisher Scientific, Darmstadt, Germany; Cat#: J60387.MC) and 0.5 mM nitroblue tetrazolium (ThermoFisher Scientific, Darmstadt, Germany; Cat#: 34035) diluted in 2% PBST for 60–90 min. The incubation was conducted in the dark at 37°C. With the same NADPH staining protocol, another set of brain sections was exclusively stained for NADPH. Finally, slices were rinsed (5 × 10 min in PBS) and mounted onto gelatin‐coated slides. They were dehydrated in ethanol and xylene and coverslipped with DPX (Fluka, Munich, Germany).

Next, we conducted fluorescent stainings to search for possible colocalization of TH and nNOS. For that, slices were first rinsed in PBS (3 × 10 min) and then incubated in 10% normal horse serum (Vectastain Elite ABC kit, Vector Laboratories, Newark, USA; Cat#: PK‐6102) in 0.3% PBST for 60 min to block unspecific binding sites. Without rinsing, slices were transferred to the primary antibody solution. The solution contained a polyclonal rabbit anti‐TH antibody (Sigma–Aldrich, St. Louis, USA; 1:2000; Cat#: AB152, RRID: AB_390204) and a polyclonal goat anti‐nNOS antibody (Invitrogen, Darmstadt, Germany; 1:1000; Cat#: OSN00004G, RRID: AB_10709561) diluted in PBST with 5% bovine serum albumin. After 72 h of incubation at 4°C, slices were rinsed in PBS (3 × 10 min) and transferred to the secondary antibody solution containing donkey anti‐rabbit AlexaFluor405 (Invitrogen, Darmstadt, Germany; 1:500; Cat#: A48258, RRID: AB_2890547) and donkey anti‐goat AlexaFluor594 (Invitrogen, Darmstadt, Germany; 1:500; Cat#: A11058, RRID: AB_2534105) diluted in PBST for 60 min at room temperature. After final rinsing in PBS (3 × 10 min), slices were mounted onto glass slides (Superfrost Plus, Thermo Scientific) and embedded with Fluoromount‐G (SouthernBiotech, Birmingham, USA). We ensured that exposure to light was reduced to a minimum in order to preserve as much fluorescence as possible.

Moreover, we conducted a triple staining against sGC, NADPH, and TH. Therefore, we started with a DAB staining against sGC, followed by a NADPH‐d staining as described above and finished with a fluorescence staining against TH. More specifically, for the sGC staining, free floating slices were stained permanently by conducting a DAB reaction with a commercially available DAB‐Kit (DAB Substrate Kit SK‐4100, Vector Laboratories, Newark, USA; Cat#: SK‐4100). Slices were first rinsed in PBS (pH 7.4; 3 × 10 min). Then, they were incubated in 0.3% H_2_O_2_ in distilled water for 30 min to block endogenous peroxidases. After that, slices were again rinsed in PBS (3 × 10 min) and incubated in 10% normal horse serum (Vectastain Elite ABC kit, Vector Laboratories, Newark, USA; Cat#: PK‐6102) in 0.3% PBST for 60 min to block unspecific binding sites. Subsequently, slices were incubated in a polyclonal rabbit anti‐sGC (FabGennix, Frisco, USA; 1:1000 in PBST; Cat#: SGCB‐201AP) for 72 h at 4°C. After that, slices were rinsed in PBS (3 × 10 min) and incubated in a secondary biotinylated donkey anti‐rabbit antibody (Jackson ImmunoResearch, Ely, UK; 1:500; Cat#: #711‐065‐152, RRID: AB_2340593) diluted in PBST for 60 min at room temperature. After further rinsing in PBS (3 × 10 min), they were incubated in an avidin–biotin complex solution (Vectastain Elite ABC kit, Vector Laboratories, Newark, USA; Cat#: PK‐6102) diluted in PBST (1:100 each) for 60 min. After that, slices were rinsed in PBS (3 × 10 min) and transferred to the DAB working solution containing two drops (50 µL) of DAB stock solution and one drop (42 µL) of buffer stock solution per 5 mL of distilled water. The staining reaction was carried out in cell wells, and each well contained 2 mL of DAB working solution. To initiate the reaction, 3 µL of H_2_O_2_ was added to each well, and after a 90 s incubation time, slices were transferred to PBS to stop the reaction. They were then rinsed in PBS (3 × 10 min) for the last time. Immediately after that, the NADPH‐d staining was carried out as described above. For the subsequent TH fluorescence staining, slices were first rinsed in PBS (3 × 10 min) and then incubated in 10% normal horse serum (Vectastain Elite ABC kit, Vector Laboratories, Newark, USA; Cat#: PK‐6102) in PBST for 60 min to block unspecific binding sites. Without rinsing, slices were transferred to the primary antibody solution containing a polyclonal rabbit anti‐TH antibody (Sigma–Aldrich, St. Louis, USA; 1:500; Cat#: AB152, RRID: AB_390204) diluted in PBST with 5% bovine serum albumin. After 72 h of incubation at 4°C, slices were rinsed in PBS (3 × 10 min) and transferred to the secondary antibody solution containing donkey anti‐rabbit AlexaFluor594 (Invitrogen, Darmstadt, Germany; 1:200; Cat#: A21207, RRID: AB_141637) diluted in PBST for 60 min at room temperature. After final rinsing in PBS (3 × 10 min), slices were mounted onto glass slides (Superfrost Plus, Thermo Scientific) and embedded with Fluoromount‐G (SouthernBiotech, Birmingham, USA). We ensured that exposure to light was reduced to a minimum in order to preserve as much fluorescence as possible.

### 2.4 Microscopic Analysis

All slices were imaged bilaterally at 100x magnification with a ZEISS Axio Scan.Z1 (9.94x/0.45 Hamamatsu Orca Flash). Images were then processed using ZEN blue 3.7 (ZEISS, Jena, Germany).

## 3 Results

### 3.1 Distribution of NO‐Synthesizing Neurons and TH

**FIGURE 1:**
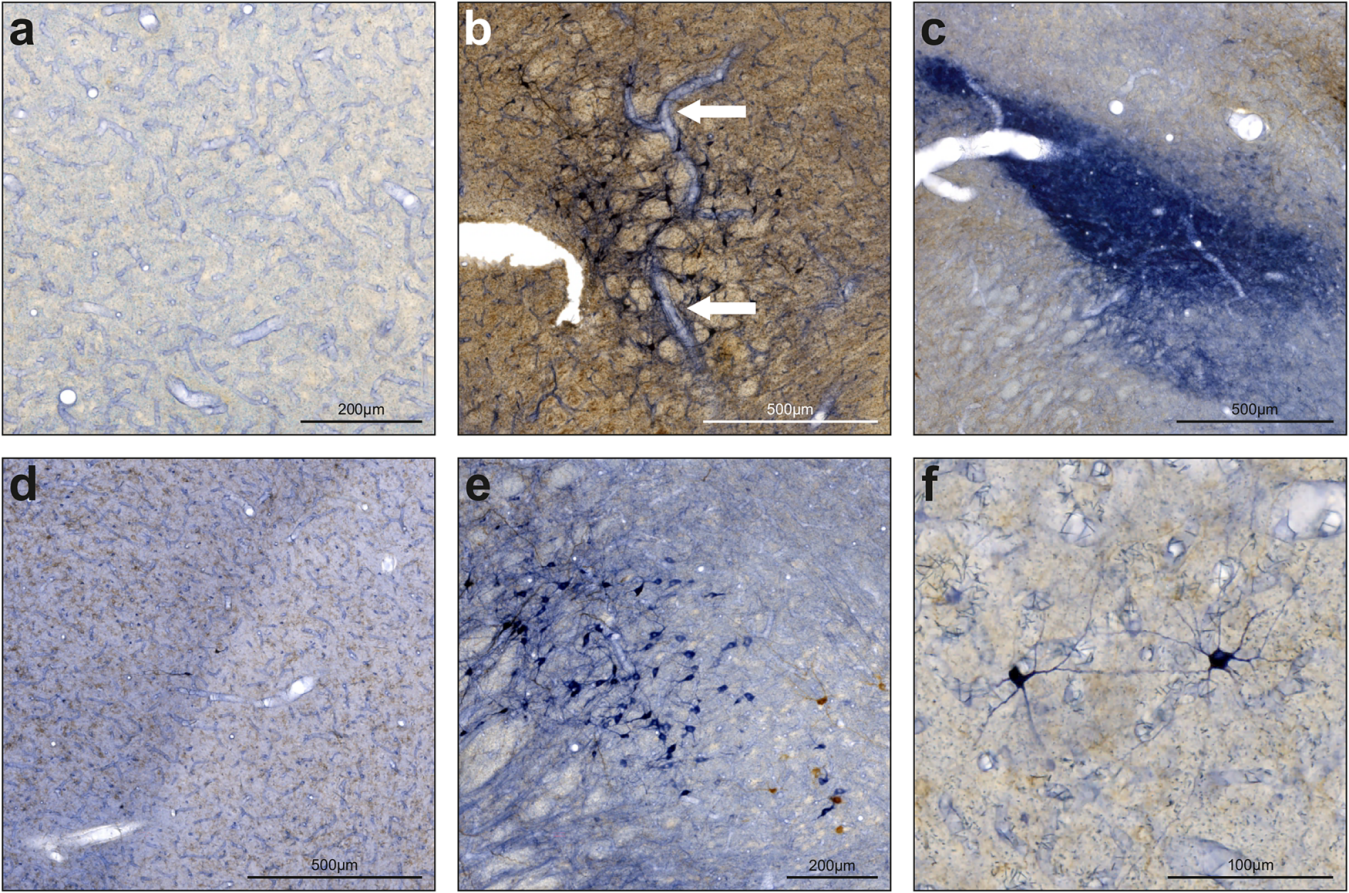
Different reaction products following NADPH‐d staining. NADPH‐d activity is illustrated in blue. In some slices, brown reaction products additionally illustrate TH immunoreactivity. If not otherwise specified, descriptions refer to the blue staining of NADPH‐d. (a) Comprehensive staining of capillaries. The example picture displays Rt. (b) Rare staining of prominent blood vessels. The white arrows indicate a vessel in the midbrain. The brown background reflects a dense dopaminergic innervation of this area. (c) Intensive neuropil staining. The example picture displays SpM. (d) Unspecific background staining. Together with staining of capillaries, this was observed throughout the whole brain. Here, the border between HA and HI is clearly visible. Additionally, dopaminergic baskets are visualized in brown. (e, f) Individually stained neurons. Somata, dendrites, and axons were usually easy to distinguish from background and TH‐positive structures, which were stained in brown. For abbreviations, see list.

**FIGURE 2:**
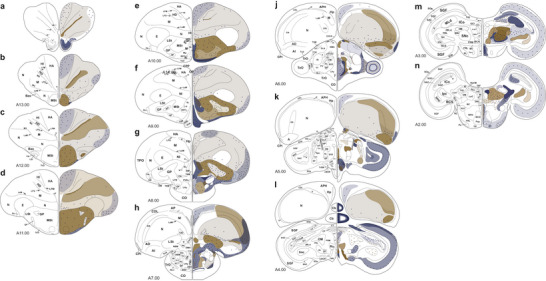
Schematic distributions of NADPH‐d activity and TH immunoreactivity. For each slice, the left side displays the specific area denotations, and the right side shows NADPH‐d and TH labeling. NADPH‐d activity is represented in blue and TH immunoreactivity in brown. Dots indicate labeled neurons, and shaded areas indicate labeled fibers and neuropil. The number of dots does not represent the true but the relative number of labeled neurons. The intensity of a shaded area represents the density of fibers and neuropil, with darker colors reflecting a higher density. For abbreviations, see Karten and Hodos (1967), Reiner et al. (2004), and list.

In order to investigate the potential role of NO in memory flexibility within the pigeon brain, we assessed the distribution of NO‐synthesizing neurons alongside dopaminergic projections, visualized by NADPH‐d activity and TH immunoreactivity (Figures 1 and 2). The distinct labeling of NADPH‐d as a marker for NO‐synthesizing neurons and TH for dopaminergic pathways allows us to observe their specific and overlapping patterns across brain regions. We utilized brain tissue from four pigeons, applying NADPH‐d and TH staining to the same slices. This dual‐staining approach revealed four prominent types of NADPH‐d labeling (Figure 1). In particular, we saw subtle, widespread capillary staining (Figure 1a,b), dense neuropil staining, often regional (Figure 1c), unspecific background staining, which was useful for delineating borders between nuclei (Figure 1d), and clear visualization of individual neurons, with distinct dendritic and axonal projections (Figure 1e,f). Most NADPH‐d‐positive neurons were easily distinguishable from TH‐positive structures, facilitating a thorough mapping of each staining type across various brain regions. All coordinates were obtained from the pigeon brain atlas by Karten and Hodos (1967).

We found NADPH‐d‐positive neurons, fibers, or neuropil exist in almost every region of the pigeon brain, indicating a very versatile and/or generalized role of NO. For better readability, we will refer to specific brain areas mostly by their abbreviated names. The most prominently labeled regions included OB, CPP, TPO, TuO, LFB, POA, SM, HM, RS, LA, GLv, GLdp, SpM, LHy, IS, ICo, MLd, nIV, MPv, IP, PM, PL, LLd, CS, nBOR, and certain layers of TeO (Figure 2). In general, NADPH‐d activity was least intense in telencephalic regions and increased toward thalamic and midbrain regions (Figure 2). However, compared to previous NADPH‐d studies, labeling in the entopallium, arcopallium, TnA, Rt, VTA, Ru, Imc, Ipc, and VLV was rather weak (Figure 2). Within TeO, we detected three bands of positive neurons. Although we investigated TH and NADPH distributions within the whole brain (Figure 2), our result section will especially focus on the telencephalon, which is the major target of TH‐positive fibers and relevant TH‐positive midbrain structures to assess the neuroanatomical framework of the memory flexibility theory.

#### 3.1.1 Telencephalon

**FIGURE 3:**
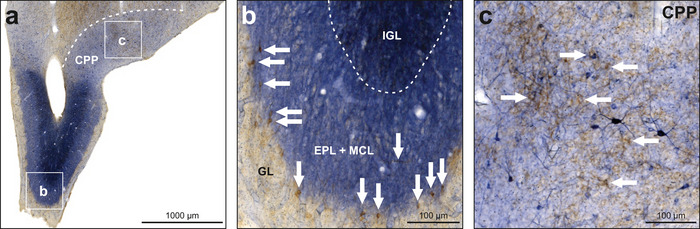
NADPH‐d and TH in the olfactory bulb. NADPH‐d is visualized in blue and TH in brown. (a) The OB displayed intense NADPH‐d activity in neuropil. (b) Enlargement of box b depicted in (a). The white arrows along the border between GL and EPL indicate TH‐positive neurons, and the arrow in EPL indicates a TH‐positive fiber. (c) Enlargement of box c depicted in (a). Within CPP, neurons and neuropil positive for NADPH‐d were detected as well as fibers and fiber baskets positive for TH. Example TH‐positive fiber baskets are indicated by white arrows. For abbreviations, see list.

The olfactory bulb showed intense NADPH‐d neuropil staining for the external plexiform layer (EPL) and mitral cell layer (MCL) and very intense staining for the internal granular layer (IGL), while the glomerular layer (GL) was spared (Figures 2a and 3a,b). There were clearly no NADPH‐d‐positive neurons in the EPL or MCL. However, detection of labeled neurons in the IGL was not possible due to the very intense neuropil staining. Few weakly TH‐positive neurons were found at the border between GL and EPL (Figure 3b), and few weakly TH‐positive fibers were found throughout all layers of the olfactory bulb. Moderate NADPH‐d neuropil staining, as well as both weakly and intensely stained neurons, was observed in the prepiriform cortex (CPP; Figures 2a and 3c). Few weakly TH‐positive fibers and fiber baskets were also present here.

**FIGURE 4:**
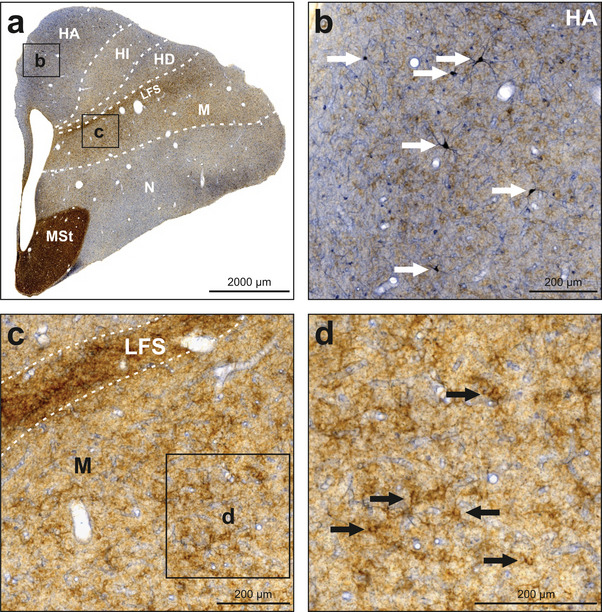
NADPH‐d and TH labeling in the hyperpallium and mesopallium. NADPH‐d is visualized in blue and TH in brown. (a) Frontal section at A13.00 stained for NADPH‐d and TH. (b) Enlargement of box b depicted in (a). HA displayed moderate NADPH‐d activity in both neuropil and neurons. White arrows indicate examples of intensely labeled neurons with axons and dendrites. Fibers and fiber baskets positive for TH are also visible. (c) Enlargement of box c depicted in (a). The mesopallium, especially the LFS, displayed a dense innervation by fibers and fiber baskets positive for TH. No NADPH‐d activity is visible. (d) Enlargement of box d depicted in (c). Prominent fiber baskets positive for TH are indicated by black arrows. For abbreviations, see list.

Within the hyperpallium, especially within the hyperpallium apicale (HA), moderate NADPH‐d activity was observed in neuropil along with a moderate number of labeled neurons, with the majority located in the medial and dorsal portions of HA (Figures 2a–g and 4a,b). Among labeled neurons, there were bigger and intensely labeled ones with extensive axons and dendrites as well as smaller and less intensely labeled ones with only weakly labeled axons and dendrites if any. Very rarely, labeled neurons were also detected in the hyperpallium intercalatum (HI) and hyperpallium densocellulare (HD). The whole hyperpallium was weakly but evenly innervated by TH‐positive fibers and baskets. Within the mesopallium, no noteworthy NADPH‐d activity was detected except for some rare solitary neurons (Figures 2a–j and 4a,c). In contrast to the hyperpallium, the mesopallium was characterized by a high density of TH‐positive fibers and baskets (Figure 4a,b). The lamina frontalis superior (LFS) constitutes the border between hyperpallium and mesopallium and displayed a very high density of TH‐positive fibers.

**FIGURE 5:**
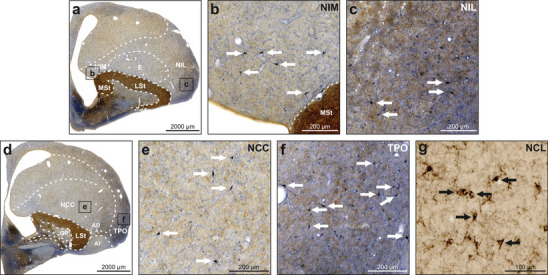
NADPH‐d and TH labeling in the nidopallium. NADPH‐d is visualized in blue and TH in brown. (a) Frontal section at A9.00 stained for NADPH‐d and TH. (b) Enlargement of box b depicted in (a). NIM displayed weakly TH‐positive fibers. White arrows indicate examples of intensely NADPH‐d‐positive neurons. (c) Enlargement of box c depicted in (a). Within NIL, a moderate density of TH‐positive and NADPH‐d‐positive fibers was observed. White arrows indicate examples of intensely NADPH‐d‐positive neurons. (d) Frontal section at A7.50 stained for NADPH‐d and TH. (e) Enlargement of box e depicted in (d). The NCC displayed a weak but even innervation by TH‐positive fibers. NADPH‐d‐positive fibers were also visible. White arrows indicate examples of intensely NADPH‐d‐positive neurons. (f) Enlargement of box f depicted in (d). TPO displayed fibers positive for NADPH‐d and TH. White arrows indicate examples of intensely NADPH‐d‐positive neurons. (g) TH‐positive fiber baskets in the NCL indicated by black arrows. Note that the depicted section was stained for TH only. For abbreviations, see list.

Within the nidopallium, NADPH‐d‐labeled neurons were detected throughout its entire anteroposterior extent (Figure 2a–l). In particular, neurons were predominantly located in the nidopallium intermedium medialis (NIM; Figure 5a,b), nidopallium intermedium laterale (NIL; Figure 5a,c), ventral nidopallium caudocentrale (NCC; Figure 5d,e), piriform cortex (CPi), and temporo‐parieto‐occipital area (TPO; Figure 5d,f). A substantial number of NADPH‐d‐positive neurons was also detected in the ventrolateral nidopallium dorsally adjacent to the arcopallium. Moderate to intense NADPH‐d staining was observed in neuropil for NIL, TPO, and CPi. Most of the nidopallium displayed a low density of TH‐positive fibers and fiber baskets except for the nidopallium caudolaterale (NCL), which displayed a very high density of TH‐positive baskets (Figure 5g).

**FIGURE 6:**
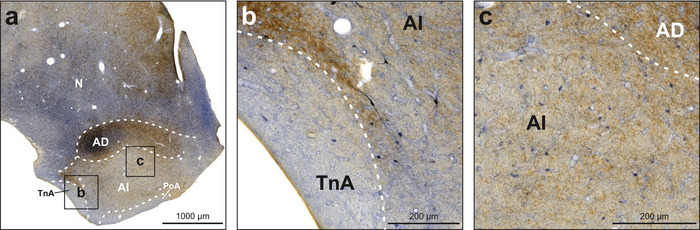
NADPH‐d and TH labeling in the arcopallium and adjacent limbic structures. NADPH‐d is visualized in blue and TH in brown. (a) Frontal section at A6.00 stained for NADPH‐d and TH. (b) Enlargement of box b depicted in (a). TnA is mostly negative for both NADPH‐d and TH, while AI displays neurons intensely and weakly positive for NADPH‐d. (c) Enlargement of box c depicted in (a). AI displayed moderate TH immunoreactivity and evenly distributed NADPH‐d‐positive neurons. For abbreviations, see list.

Within the arcopallium, we observed weak NADPH‐d activity in neuropil in the dorsal arcopallium (AD), increasing toward its medial portion (Figure 6a). We did not observe NADPH‐d‐positive neuropil in the intermediate arcopallium (AI; Figure 6a,c). Labeled neurons were found across all subdivisions and were mostly small and weakly stained (Figures 2h–l and 6c). Few intensely stained neurons were scattered. While AD was heavily innervated by TH‐positive fibers, AI showed only weak to moderate TH immunoreactivity (Figures 2h–l and 6a,c). The posterior arcopallium also showed TH immunoreactivity in ventral AI. The adjacent nucleus taeniae of the amygdala (TnA) and posterior pallial amygdala (PoA) were in general negative for both TH and NADPH‐d (Figure 6a,b). In some animals, we detected small neurons that were faintly positive for NADPH‐d in PoA. In some animals, the border between TnA and AI was positive for either TH or NADPH‐d or both, and in some animals, we also observed NADPH‐d‐positive neurons in this border region.

**FIGURE 7:**
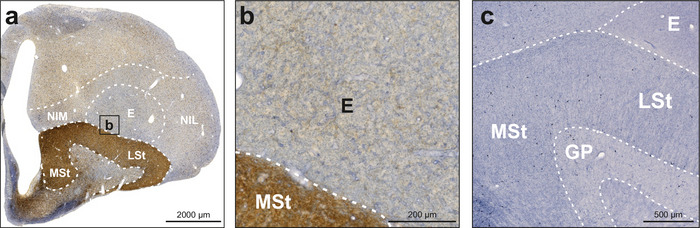
NADPH‐d and TH labeling in the entopallium and adjacent basal ganglia. NADPH‐d is visualized in blue and TH in brown. (a) Frontal section at A9.50 stained for NADPH‐d and TH. (b) Enlargement of box b depicted in (a). The entopallium showed weak immunoreactivity for TH but was negative for NADPH‐d. (c) Frontal section at A9.50 stained for NADPH‐d. The striatum showed moderate immunoreactivity for NADPH‐d. For abbreviations, see list.

The entopallium did not show NADPH‐d activity in neuropil, and labeled neurons were detected very rarely (Figures 2d–g and 7a,b). TH‐positive fibers and fiber baskets were very sparse but evenly distributed. The striatum was heavily innervated by TH‐positive fibers so that the NADPH‐d staining was hard to assess. Therefore, we also stained some sections exclusively against NADPH‐d to investigate neuropil and neurons in these regions. We found a moderate number of NADPH‐d‐positive neurons in the medial striatum (MSt), lateral striatum (LSt), and globus pallidus (GP) (Figure 7c).

**FIGURE 8:**
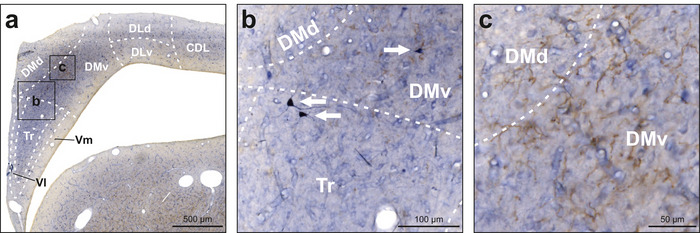
NADPH‐d and TH labeling in the hippocampus. NADPH‐d is visualized in blue and TH in brown. (a) Frontal section at A5.00 stained for NADPH‐d and TH. (b) Enlargement of box b depicted in (a). NADPH‐d‐positive neuropil was observed in Tr, DMd, and DMv. White arrows indicate examples of intensely NADPH‐d‐positive neurons. (c) Enlargement of box c depicted in (a). In DMd and DMv, intensely TH‐positive fibers were detected. For abbreviations, see list.

Within the hippocampus, neuropil was moderately positive for NADPH‐d, particularly in the triangular region (Tr), and to a lesser extent also in the dorsal and ventral portions of the dorsomedial area (DMd, DMv; Figures 2h–l and 8a,b). NADPH‐d‐positive neurons were found in all hippocampal subregions as well as in the area corticoidea dorsolateralis (CDL; Figures 2h and 8a). Moreover, we detected a cluster of intensely stained TH‐positive fibers located in DMd that extended into the dorsal portion of DMv in some animals (Figure 8c). In some animals, this cluster also contained few TH‐positive neurons.

**FIGURE 9:**
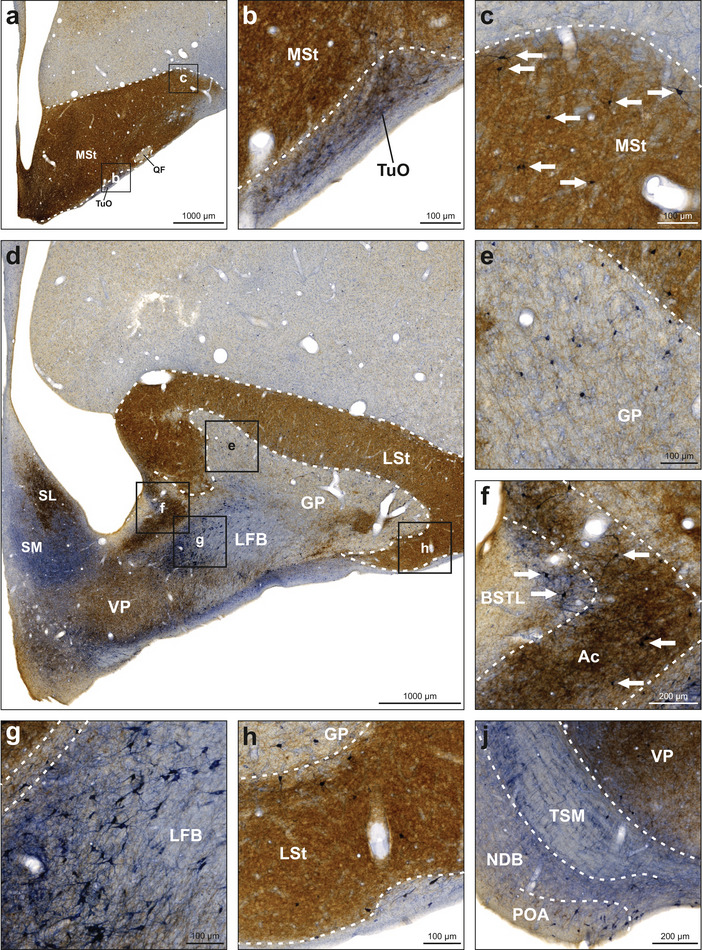
NADPH‐d and TH labeling in the basal ganglia and adjacent structures. NADPH‐d is visualized in blue and TH in brown. (a) Frontal section at A10.50. (b) Enlargement of box b depicted in (a). Fibers in the TuO were positive for both NADPH‐d and TH. Neurons weakly positive for NADPH‐d were also detected. (c) Enlargement of box c depicted in (a). The MSt was heavily innervated by TH‐positive fibers. White arrows indicate neurons intensely positive for NADPH‐d. (d) Frontal section at A8.00. (e) Enlargement of box e depicted in (d). The GP contained a moderate number of NADPH‐d‐positive neurons, and fibers positive for both NADPH‐d and TH. (f) Enlargement of box f depicted in (d). The Ac was heavily innervated by TH‐positive fibers and exhibited few NADPH‐d‐positive neurons as indicated by white arrows. The dorsolateral portion of the adjacent BSTL displayed a thin band of NADPH‐d‐positive fibers and neurons as indicated by white arrows. (g) Enlargement of box g depicted in (d). The dorsomedial portion of the LFB showed intensely NADPH‐d‐positive neurons and fibers. (h) Enlargement of box h depicted in (d). The LSt was heavily innervated by TH‐positive fibers and contained a moderate number of NADPH‐d‐positive neurons. (j) Frontal section at A9.00. The VP was heavily innervated by TH‐positive fibers. The TSM showed NADPH‐d activity in fibers running along the tract as well as in fibers crossing orthogonally. NDB showed fibers positive for NADPH‐d and TH, and NADPH‐d‐positive neurons in the ventrolateral portion. POA contained a high number of NADPH‐d‐positive neurons and fibers positive for NADPH‐d and TH. For abbreviations, see list.

Within the basal ganglia, both LSt and MSt contained a moderate number of NADPH‐d‐positive neurons, with the majority located at the periphery toward the nidopallium, entopallium, or GP (Figures 2, 7, and 9c,h). The GP also contained a moderate number of intensely NADPH‐d‐positive neurons (Figures 2f,g and 9d,e). In some animals, these were primarily located in the dorsal portion along the border between GP and LSt. GP fibers were positive for both NADPH‐d and TH. The lateral forebrain bundle (LFB) displayed weakly NADPH‐d‐positive fibers in the dorsolateral portion and intensely positive fibers in the ventromedial portion. We also observed a cluster of intensely labeled neurons along the medial border of the LFB (Figure 9d,g). Moreover, we observed intense neuropil staining in the adjacent nucleus septalis medialis (SM), nucleus preopticus anterior (POA), and tuberculum olfactorium (TuO). In the POA and TuO, we also detected labeled neurons. The nucleus of the diagonal band (NDB) displayed fibers positive for both NADPH‐d and TH (Figure 9j). The nucleus accumbens (Ac) was heavily innervated by TH‐positive fibers and also displayed few NADPH‐d‐positive neurons. In the dorsal portion of the lateral bed nucleus of the stria terminalis (BSTL), we observed a small band of NADPH‐d‐positive neurons and neuropil (Figure 9d,f). Furthermore, TH‐positive fibers were found in the TuO, POA, ventral LFB, ventral pallidum (VP), nucleus septalis lateralis (SL), nucleus commissuralis septi (CoS), and tractus cortico‐habenularis et cortico septalis (CHCS). Because of the heavy TH immunoreactivity, it was very difficult to detect any potential NADPH‐d activity in Ac or SL.

#### 3.1.2 Midbrain

**FIGURE 10:**
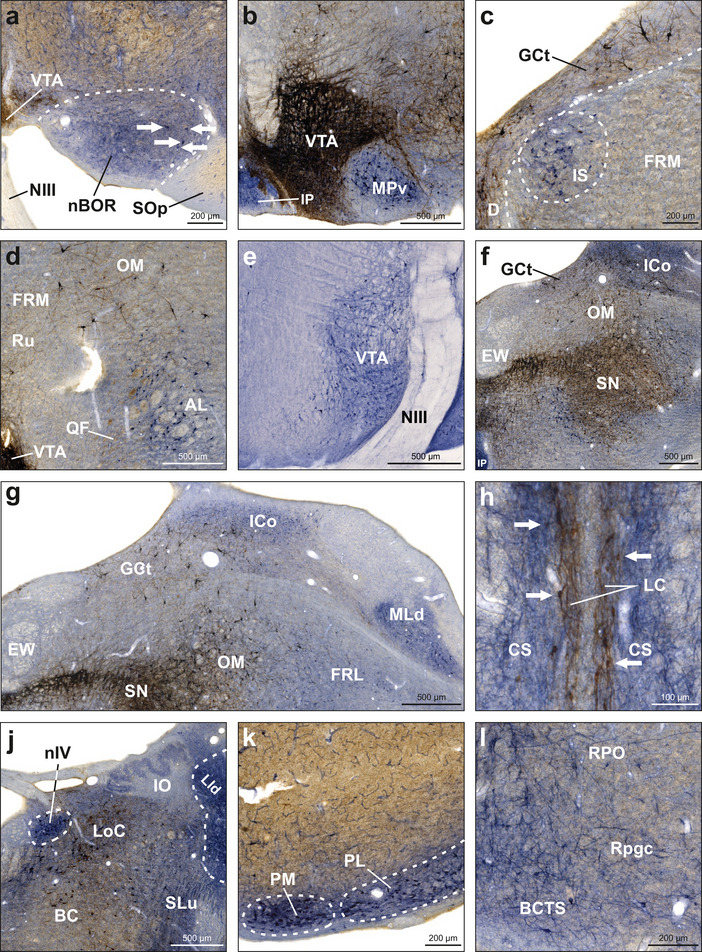
NADPH‐d and TH labeling in the midbrain. NADPH‐d is visualized in blue and TH in brown. All pictures depict right hemispheres except for (H), which depicts a section along the midline of the slice including both hemispheres. (a) Frontal section at A4.00. Neuropil in the nBOR was moderately positive for NADPH‐d. White arrows indicate labeled neurons. (b) Frontal section at A3.50. VTA contained a dense network of TH‐positive neurons and fibers. NADPH‐d activity in the VTA was also observed but difficult to distinguish due to the strong TH staining intensity. Neuropil and neurons in IP and MPv were NADPH‐d‐positive. (c) Frontal section at A4.00. IS contained neuropil and neurons positive for NADPH‐d. D and GCt showed TH‐positive neurons and fibers. FRM contained weakly TH‐positive fibers and weakly NADPH‐d‐positive neurons. (d) Frontal section at A4.00. VTA contained a dense network of TH‐positive neurons and fibers. QF showed weakly TH‐positive fibers. Intensely NADPH‐d‐positive neurons were distributed across AL and adjacent areas. Ru contained TH‐positive fibers. Intensely TH‐positive neurons were distributed across OM. (e) Frontal section showing NADPH‐d‐positive neurons within VTA. Note that this panel displays a section stained for NADPH‐d only. (f) Frontal section at A3.00. SN was intensely TH positive in neurons and fibers. NADPH‐d‐positive neurons were detected in the ventral SN and extended beyond its medial border. OM displayed TH‐positive neurons and fibers extending into GCt and ventral ICo. ICo also showed NADPH‐d activity in neurons and neuropil. (g) Frontal section at A3.00. Dorsal ICo showed NADPH‐d activity in neuropil and neurons as well as TH‐positive fibers. Ventral ICo predominantly contained TH‐positive neurons and fibers. MLd showed NADPH‐d activity in neuropil, neurons, and fibers in its ventrolateral tip. (h) Frontal section at A2.25. LC contained TH‐positive fibers and neurons. White arrows indicate examples of labeled neurons. CS contained NADPH‐d‐positive fibers. In other cases, we also observed NADPH‐d‐positive neurons. (j) Frontal section at A2.00. LoC displayed TH immunoreactivity in neuropil, neurons, and fibers, and NADPH‐d activity in neurons and fibers. nIV was intensely NADPH‐d‐positive. TH‐positive neurons and fibers were distributed across BC. Neuropil in LLd was intensely NADPH‐d‐positive. SLu showed NADPH‐d activity in neuropil and neurons and few TH‐positive fibers. (k) Frontal section at A1.75. PM and PL contained intensely NADPH‐d‐positive neuropil and a high number of labeled neurons. (l) Frontal section at A1.50. A network of NADPH‐d‐positive fibers and few neurons was distributed across RPO, RPgc, and BCTS. For abbreviations, see list.

Within the midbrain, numerous nuclei were found positive for either NADPH‐d or TH or both (Figure 2l–n). The nucleus of the basal optic root (nBOR) displayed weak to moderate NADPH‐d activity in neuropil and only very few neurons (Figure 10a). Neurons and fibers in the VTA were intensely positive for TH (Figure 10a,b,d). Since the TH staining was so strong in VTA, it was hard to assess NADPH‐d labeling. Therefore, we selectively stained some sections against NADPH‐d and found a large amount of NADPH‐d‐positive neurons within VTA (Figure 10e). Medial to the nervus occulomotorius (NIII) and dorsal to the nucleus interpeduncularis (IP), we detected more TH‐positive fibers. IP contained intensely NADPH‐d‐positive neuropil and some neurons (Figure 10b,f). The nucleus ruber (Ru) contained TH‐positive fibers but was negative for NADPH‐d (Figure 10d). The nucleus interstitialis (IS) was easily distinguishable from its surroundings based on weak NADPH‐d staining in neuropil and a moderate number of labeled neurons (Figure 10c). Occasionally, we detected TH‐positive neurons and fibers within the medial portion of IS, but it is likely that these belong to adjacent nucleus Darkschewitsch (D). The substantia grisea centralis (GCt) mainly contained TH‐positive neurons and fibers, but we also observed weakly NADPH‐d‐positive neuropil and few neurons (Figure 10c,f,g). The tractus quintofrontalis (QF) exhibited weakly TH‐positive fibers (Figure 10d). Intensely NADPH‐d‐positive neurons were found in ansa lenticularis (AL) extending into the formation reticularis lateralis mesencephalic (FRL) and the tractus occipitomesencephalicus (OM), which predominantly contained TH‐positive fibers and neurons (Figure 10d). The formation reticularis medialis mesencephalic (FRM) mainly contained weakly NADPH‐d‐positive neurons and weakly TH‐positive fibers (Figure 10c,d). The nucleus mesencephalicus lateralis, pars dorsalis (MLd) showed an interesting pattern of NADPH‐d activity. In none of the animals, the whole nucleus showed reactivity. In the anterior portion of MLd, we observed two discrete clusters of NADPH‐d activity. The ventral cluster was usually bigger with more intense staining in neuropil and neurons, whereas the smaller dorsal cluster only showed activity in neuropil. In the posterior portion of MLd, activity was mainly concentrated at the ventrolateral tip and along the dorsal and ventral borders of the nucleus (Figure 10g). The nucleus intercollicularis (ICo) showed moderate NADPH‐d activity in neuropil and neurons, especially in its posterior portion. In its intermediate portion, where ICo is located between GCt and OM, we also detected many TH‐positive neurons and fibers in its ventral half (Figure 10f,g). The nucleus papilloformis (Pap) contained many moderately NADPH‐d‐positive neurons and fibers and also TH‐positive fibers and few neurons. The nucleus mesencephalicus profundus, pars ventralis (MPv) contained TH‐positive fibers and big intensely NADPH‐d‐positive neurons (Figure 10b). As expected and similar to the VTA, neurons and fibers in the substantia nigra (SN) were heavily positive for TH, also encompassing adjacent areas (Figure 10f,g). We also observed NADPH‐d activity in neurons and fibers throughout the SN. Some neurons and the neuropil of the nucleus centralis superior (CS) were intensely positive for NADPH‐d (Figure 10h). The nucleus linearis caudalis (LC), which is located medially to the CS, displayed a thin band of TH‐positive fibers and neurons (Figure 10h). Alongside VTA and SN, the locus ceruleus (LoC) was the third major structure that contained intensely TH‐positive neurons and fibers (Figure 10j). Beneath this dense web, we also spotted NADPH‐d‐positive neurons. The brachium conjunctivum descendens (BCD) displayed a network of NADPH‐d‐positive fibers but no neurons. Furthermore, the nucleus lemnisci lateralis, pars dorsalis (LLd) showed very intense NADPH‐d activity in neuropil (Figure 10j). The nucleus isthmo‐opticus (IO) showed weak to moderate NADPH‐d activity in neuropil (Figure 10j). The nucleus nervi trochlearis (nIV) displayed a dense network of NADPH‐d‐positive fibers, neuropil, and neurons (Figure 10j). The nucleus also contained TH‐positive fibers. In some, but not all, birds, we observed NADPH‐d activity in neuropil in the nucleus semilunaris (SLu; Figure 10j). The nucleus ventralis lemnisci lateralis (VLV) was negative for both NADPH‐d and TH. The nucleus pontis lateralis (PL) and nucleus pontis medials (PM) in the ventral midbrain both showed moderate NADPH‐d activity in neuropil. In PM, a high number of NADPH‐d‐positive neurons clustered, whereas in PL, a moderate number of neurons was distributed throughout the nucleus (Figure 10k). A network of NADPH‐d‐positive neurons and fibers was widely distributed across the brachium conjunctivum descendens et tractus tectospinalis (BCTS), nucleus reticularis pontis oralis (RPO), nucleus reticularis pontis caudalis, pars gigantocellularis (RPgc), and corpus trapezoideum (Figure 10l).

### 3.2 Colocalization of nNOS and TH

Thus far, we investigated the distributions of NADPH‐d and TH separately, which did not allow for a precise detection of colocalizations. However, in order to assess the neuroanatomical framework of the memory flexibility theory that has been established in fruit flies, we needed a combined investigation of TH, nNOS, and sGC. We therefore conducted fluorescence stainings with tissue of six animals to determine where TH and nNOS overlap within the midbrain and whether TH‐ and nNOS‐positive fibers target sGC‐positive cells in the forebrain.

**FIGURE 11:**
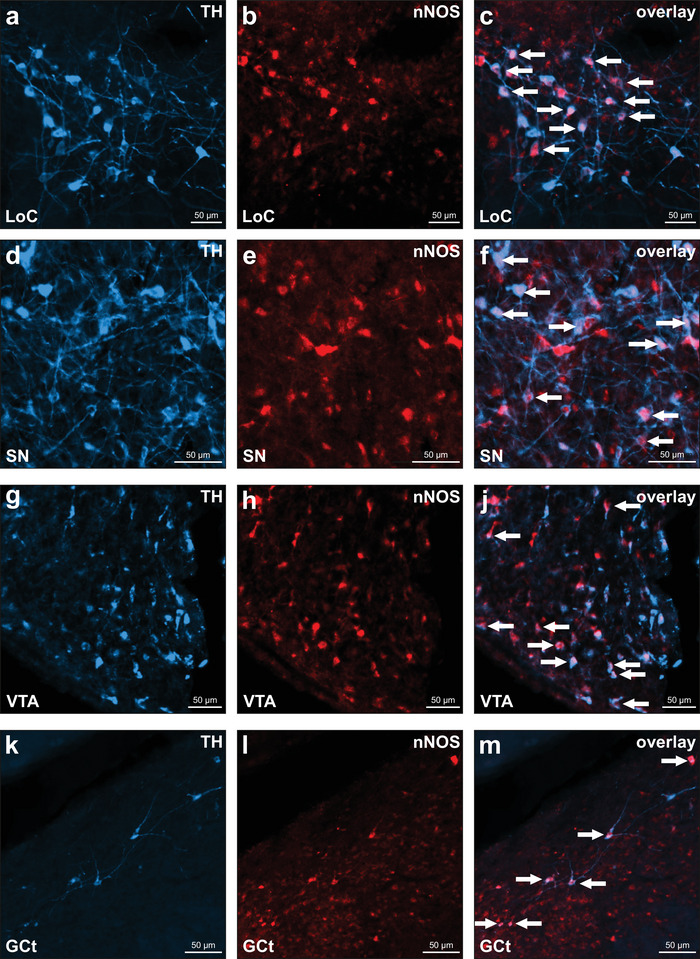
Colocalization of nNOS and TH. Each row is dedicated to one TH‐positive midbrain structure (from top to bottom: LoC, SN, VTA, GCt). Left column pictures depict TH‐positive neurons in blue, middle column pictures depict nNOS‐positive neurons in red at the same site, and right column pictures depict overlays. White arrows indicate exemplary neurons positive for both TH and nNOS. For abbreviations, see list.

All of the major TH‐positive midbrain structures—LoC, VTA, and SN—showed comparable colocalization of TH and nNOS (Figure 11a–j). In addition, we found that many TH‐positive neurons in the GCt also expressed nNOS (Figure 11k–m).

**FIGURE 12:**
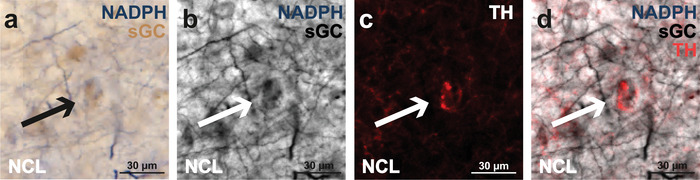
Spatial association of TH‐, NADPH‐d‐, and sGC‐positive neurons and fibers in the NCL using triple staining. (a) NADPH‐d‐positive fibers are displayed in blue and sGC‐positive neurons in brown. (b) The same region is presented in grayscale, showing NADPH‐d and sGC staining without color. (c) Fluorescent staining shows TH‐positive fibers in red, highlighting their distribution in the NCL. (d) Overlay of red TH fluorescence from (c) and the grayscale image from (b), demonstrating how fibers positive for both NADPH‐d and TH appear to encircle the sGC‐positive neurons. For abbreviations, see list.

Furthermore, we investigated the anatomical relationships among TH‐ and NADPH‐d‐positive afferent fibers and sGC‐positive cells in the pigeon NCL, which is one of the primary targets of dopaminergic input in the avian telencephalon and commands executive functions (Figure 2). Therefore, we conducted a triple staining protocol, in which sGC‐positive cells were permanently stained brown using our DAB staining protocol (Figure 12a,b), NO‐synthesizing fibers were labeled blue through NADPH‐d staining (Figure 12a,b), and TH‐positive fibers were labeled red using fluorescence staining (Figure 12c). Our results reveal that both NADPH‐d‐ and TH‐positive fibers appear to encircle sGC‐positive cells, indicating a close spatial association (Figure 12d).

## 4 Discussion

This study provides new insights into the distribution of NO‐synthesizing neurons in the pigeon brain and their potential interactions with dopaminergic pathways, particularly in relation to memory flexibility. By mapping NADPH‐d, nNOS, sGC, and TH, we identified a neuroanatomical framework supporting NO–dopamine interactions. These findings support the view that NO might play a role in modulating cognitive processes such as memory adaptation, complementing dopamine's established functions in reinforcement learning. In particular, the structural convergence of NO and dopamine markers in the NCL suggests a potential mechanism for regulating the balance between memory consolidation and forgetting, a feature reminiscent of NO's function in *D. melanogaster* (Aso et al. 2019).

### 4.1 NO and Memory Flexibility

**FIGURE 13:**
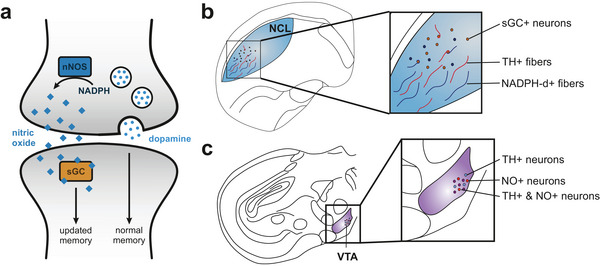
Summary of the memory flexibility hypothesis and our main findings. (a) In *Drosophila melanogaster*, a subset of dopaminergic neurons releases both dopamine and NO, enabling the fly to both reinforce and gradually “forget” memories associated with rewards or punishments (Aso et al. 2019; Green and Lin 2020). In this system, dopamine strengthens memory traces tied to meaningful experiences, while NO serves as a complementary, slowly acting signal that weakens these memories over time. (b) In pigeons, a similar neuroanatomical framework can be found as some TH‐ and NADPH‐d‐positive fibers terminate on sGC‐positive neurons within the NCL, while (c) some neurons within the VTA express both NO and TH. For abbreviations, see list.

Memory is a dynamic process, requiring both stabilization and modification of existing traces. NO has been implicated as a key modulator of memory flexibility in *D. melanogaster*, where it co‐releases with dopamine in select neurons, promoting gradual memory decay while allowing new learning to take place (Aso et al. 2019; Green and Lin 2020). Experimental work in *D. melanogaster* has demonstrated that NO‐producing dopaminergic neurons in the mushroom body regulate memory stability by weakening associations over time. Aso et al. (2019) found that genetic inhibition of NO synthase in *D. melanogaster* led to an overstabilization of memories, preventing their expected decay, while NO signaling facilitated memory updating and reduced retention duration (Figure 13a). This indicates that NO functions as a regulatory signal, ensuring that memories do not persist indefinitely but instead remain adaptable to environmental changes. In our study, we show that within the NCL, both dopaminergic projections and NADPH‐d‐positive fibers converge on the same sGC‐positive neurons (Figures 12 and 13b). Building on findings from *D. melanogaster*, this structural arrangement suggests that NO in pigeons may also contribute to the modification of previously learned associations, which would be particularly relevant in extinction learning paradigms that support adaptive behavior (Packheiser et al. 2021).

The interaction between NO and dopamine is further supported by our observation that nNOS‐positive neurons are present in the VTA, LoC, and SN, with no significant difference in their colocalization with TH across these regions (Figure 13c). This implies that NO may influence multiple dopaminergic pathways, aligning with previous studies in mammals where NO affects synaptic plasticity and dopamine release in various regions, including the prefrontal cortex (Bon and Garthwaite 2003; Kiss et al. 1999; Pogun et al. 1994; Trabace et al. 2007; West et al. 2002). Overall, the importance of NO in regulating synaptic plasticity (Böhme et al. 1993; Bon and Garthwaite 2003), along with its presence in areas associated with decision‐making and response adaptation (Kiss et al. 1999; Trabace et al. 2007; Zoubovsky et al. 2011), supports the idea that NO plays a fundamental role in modifying pre‐existing behavioral strategies based on environmental demands.

Dopaminergic pathways are essential for working memory, reinforcement learning, cognitive flexibility, and decision‐making (Karakuyu et al. 2007; Packheiser et al. 2021; Puig et al. 2014; Pusch et al. 2023; Rook et al. 2023). In pigeons, the NCL is a primary target for dopaminergic input, with TH‐positive fibers coiling around nondopaminergic neurons (Divac et al. 1985; Kitt and Brauth 1986; Waldmann and Güntürkün 1993; Wynne and Güntürkün 1995). On the functional level, the NCL supports short‐term storage and manipulation of information, encoding stimulus–action–outcome associations and enabling cognitive flexibility such as reversal learning, extinction, and memory updating (Diekamp et al. 2002; Güntürkün 1997; Johnston et al. 2019; Veit et al. 2017). Hence, the convergence of NO‐synthesizing and TH‐positive fibers on sGC‐expressing neurons in the NCL (Figure 13b) reinforces the idea that NO may modulate dopamine‐driven cognitive functions in birds as well. Comparable mechanisms have been described in mammals, where NO‐mediated changes in prefrontal dopamine release affect working memory and learning (Trabace et al. 2007; Zoubovsky et al. 2011). Moreover, research in rodents has shown that NO acts as a neuromodulator of learning and memory processes, particularly those requiring adaptation to new contingencies. Evidence from both inhibitory avoidance and spatial learning tasks suggests that NO plays a role in memory updating, behavioral flexibility, and reinforcement‐based learning mechanisms (Harooni et al. 2009; Yamada et al. 1995). Inhibitory avoidance paradigms have demonstrated that modulation of NO in the hippocampus affects both short‐ and long‐term memory retention, indicating a role in regulating long‐term memory stability (Harooni et al. 2009). Likewise, NO inhibition impairs performance during the acquisition phase of the radial arm maze—a task requiring animals to integrate spatial cues and to avoid revisiting previously explored locations—revealing a role in spatial working memory and adaptive strategy use (Yamada et al. 1995). Furthermore, NO inhibition has been shown to alter dopamine metabolism in the striatum, implying that NO interacts with dopamine to modulate reinforcement‐based learning mechanisms (West et al. 2002). Together, these findings highlight NO as a conserved neuromodulator involved in regulating synaptic plasticity and shaping adaptive learning processes across species, with our results suggesting a similar role in the avian brain.

### 4.2 NO in Spatial Memory and Aging

Beyond its role in memory flexibility, NO has also been implicated in spatial memory formation and navigation (Estall et al. 1993; Majlessi et al. 2008; Noda et al. 1997; Yamada et al. 1995; Zou et al. 1998). The presence of NO‐synthesizing neurons in the pigeon hippocampus is consistent with the idea that NO contributes to spatial learning, as has been shown in rodents, where NO is essential for hippocampus‐dependent navigation tasks (Majlessi et al. 2008; Zhang et al. 1998). As pigeons rely heavily on spatial memory for homing (Herold et al. 2015; Strasser et al. 1998), NO may serve as a regulatory factor ensuring that spatial representations remain adaptable over time. In light of these findings, it would be valuable to investigate whether similar effects occur in birds by testing the impact of NO inhibition on spatial task performance.

In addition to its role in learning, NO has been linked to aging‐related cognitive decline (Domek‐Łopacińska and Strosznajder 2010; Law et al. 2001; McCann 1997). In mammals, a reduction in NO levels has been associated with impaired synaptic plasticity and decreased cognitive function (Domek‐Łopacińska and Strosznajder 2010). Rodent studies have shown that aged rats exhibit a reduced number of NADPH‐d‐positive neurons in the cerebral cortex and striatum, as well as reduced nNOS activity in the cerebellum. These age‐related neurochemical changes were associated with impairments in radial arm maze performance, suggesting a decline in NO‐mediated mechanisms of spatial learning (Yamada et al. 1996). Although little is known about NO's role in aging birds, evidence from zebra finches reveals that NO expression decreases in song nuclei with age, affecting song plasticity (Wallhäusser‐Franke et al. 1995). Given pigeons’ long lifespan and sustained cognitive abilities, investigating NO's potential contribution to age‐related memory decline could provide insights into its broader neuromodulatory functions.

### 4.3 Evolutionary and Comparative Perspectives

The widespread presence of NADPH‐d across avian brain regions indicates that NO plays a general modulatory role in sensory and cognitive processing, with species‐specific variations likely reflecting ecological adaptations. Studies in quails, chickens, and pigeons have demonstrated a broadly conserved NO distribution across forebrain, midbrain, and hindbrain structures (Atoji et al. 2001; Brüning 1993; Panzica et al. 1994). However, distinct ecological and behavioral specializations may influence NO expression in key regions. For example, species‐specific differences have been observed, such as stronger NO expression in the pigeon hippocampus and olfactory bulb compared to non‐homing species (Brüning 1993; Panzica et al. 1994). This suggests a potential link between NO signaling and spatial cognition, where pigeons, known for their exceptional navigational abilities (Gagliardo et al. 1999; Rook et al. 2023), might rely on NO‐mediated plasticity to process and adapt spatial information. Supporting this notion, rodent studies have shown that NO regulates hippocampal plasticity and spatial memory, with NO inhibition leading to impairments in learning tasks, such as the Morris water maze (Harooni et al. 2009; Majlessi et al. 2008). Similarly, food‐storing birds exhibit elevated NO expression in the hippocampus, likely supporting the greater spatial memory demands required for efficient retrieval of cached food (Krebs et al. 1996).

Conversely, in species that rely heavily on vocal communication, such as songbirds and parrots, NO expression is particularly elevated in auditory‐processing regions (Brauth et al. 2005; Cozzi et al. 1996; Wallhäusser‐Franke et al. 1995). In vocal learners, NO has been detected in song‐control nuclei, including HVC (acronym used as proper name), the robust nucleus of the arcopallium, and Area X, with species‐specific variations suggesting adaptations to distinct vocal learning strategies (Brauth et al. 2005; Cozzi et al. 1996). Among these species, budgerigars exhibit a higher density of NADPH‐d‐positive neurons in the anterior forebrain, particularly in the nucleus basalis, a region associated with vocal learning and auditory processing in this species (Cozzi et al. 1996). In contrast, zebra finches show an age‐dependent decline in NADPH‐d expression within song nuclei, potentially linked to differences between lifelong learners (e.g., budgerigars) and species with restricted vocal learning periods (e.g., zebra finches) (Brauth et al. 2005; Wallhäusser‐Franke et al. 1995). These findings support the idea that NO expression in song‐related circuits may be shaped by species‐specific vocal learning demands and plasticity mechanisms.

In pigeons, NO activity was detected in the arcopallium and entopallium, brain regions associated with motor control and visual processing (Atoji et al. 2001). However, NO expression in these areas appeared weaker than previously reported by Atoji et al. (2001), raising questions about potential methodological differences or individual variability. While the functional significance of this variation remains uncertain, comparative analyses suggest that NO expression patterns across species may be closely linked to distinct sensory, motor, and cognitive specializations.

Future research should explore whether NO‐mediated memory flexibility is shaped by ecological pressures, particularly in species with higher cognitive plasticity. If NO plays a key role in adaptive forgetting, comparative studies across avian taxa could reveal whether this mechanism is more pronounced in species requiring high behavioral flexibility. Further experimental work involving NO manipulation during extinction learning paradigms may provide deeper insights into how NO contributes to memory regulation and adaptive decision‐making across vertebrates.

## 5 Conclusion

This study provides anatomical evidence that NO contributes to dopaminergic modulation in the pigeon NCL, potentially supporting mechanisms that balance memory retention and forgetting. Its presence in the hippocampus and links to aging further underscore its broader relevance to avian cognition. Behavioral studies are now needed to test whether NO influences memory persistence and cognitive flexibility in birds. By establishing this neuroanatomical framework, our findings advance the understanding of NO as a conserved neuromodulatory factor in memory processing across species.

## Author Contributions

**Alina Steinemer**: conceptualization, data curation, data analysis, writing – original draft, visualization. **Marie Ziegler**: data curation, visualization. **Kevin Haselhuhn**: data curation, data analysis. **Onur Güntürkün**: conceptualization, writing – editing, funding acquisition, resources. **Noemi Rook**: conceptualization, data curation, data analysis, visualization, writing – editing, supervision.

## Conflicts of Interest

The authors declare no conflicts of interest.

## Peer Review

The peer review history for this article is available at https://publons.com/publon/10.1002/cne.70095.

## Data Availability

Data will be made available upon request from the corresponding author.
